# Interaction between thrombin potential and age on early clinical outcome in patients hospitalized for COVID-19

**DOI:** 10.1007/s11239-021-02497-1

**Published:** 2021-06-10

**Authors:** Marco G. Mennuni, Roberta Rolla, Leonardo Grisafi, Enrico G. Spinoni, Andrea Rognoni, Veronica Lio, Luigi M. Castello, Pier P. Sainaghi, Mario Pirisi, Gian Carlo Avanzi, Marco Krengli, Mattia Bellan, Daniela Ferrante, Gianluca Aimaretti, Umberto Dianzani, Giuseppe Patti

**Affiliations:** 1 Dipartimento Di Medicina Traslazionale, Università del Piemonte Orientale, Azienda Ospedaliero-Universitaria Maggiore Della Carità, Via Solaroli 17, 28100 Novara, NO Italy; 2 Università del Piemonte Orientale, Novara, Italy

**Keywords:** Bleeding, COVID-19, Thrombotic complications, Thrombin potential

## Abstract

Patients with Coronavirus Disease-2019 (COVID‐19) have haemostatic dysfunction and are at higher risk of thrombotic complications. Although age is a major risk factor for outcome impairment in COVID-19, its impact on coagulative patterns here is still unclear. We investigated the association of Endogenous Thrombin Potential (ETP) with thrombotic and haemorrhagic events according to different ages in patients admitted for COVID-19. A total of 27 patients with COVID‐19-related pneumonia, without need for intensive care unit admission or mechanical ventilation at hospital presentation, and 24 controls with non-COVID-19 pneumonia were prospectively included. ETP levels were measured on admission. Patients were evaluated for major adverse cardiovascular events (MACE: cardiovascular death, myocardial infarction, stroke, transient ischemic attack, venous thromboembolism) and bleeding complications [according to Bleeding Academic Research Consortium (BARC) definition] during in-hospital stay. COVID-19 patients had similar ETP levels compared to controls (AUC 93 ± 24% vs 99 ± 21%, p = 0.339). In the COVID-19 cohort, patients with in-hospital MACE showed lower ETP levels on admission vs those without (AUC 86 ± 14% vs 95 ± 27%, p = 0.041), whereas ETP values were comparable in patients with or without bleeding (AUC 82 ± 16% vs 95 ± 26%, p = 0.337). An interaction between age and ETP levels for both MACE and bleeding complications was observed, where a younger age was associated with an inverse relationship between ETP values and adverse event risk (p_int_ 0.018 for MACE and 0.050 for bleeding). Patients with COVID-19 have similar thrombin potential on admission compared to those with non-COVID-19 pneumonia. In younger COVID-19 patients, lower ETP levels were associated with a higher risk of both MACE and bleeding.

**Supplementary Information:**

The online version contains supplementary material available at 10.1007/s11239-021-02497-1.

## Highlights

- Coronavirus Disease-2019 (COVID-19) has haemostatic dysfunction and age is a major risk factor for adverse outcome
- Endogenous thrombin potential levels and thrombotic/haemorrhagic events were evaluated according to different ages in patients admitted for COVID-19
- Patients with Coronavirus Disease-2019 have similar thrombin potential on admission compared to those with non-COVID-19 pneumonia.
- In younger COVID-19 patients, lower endogenous thrombin potential levels were associated with a higher risk of both thrombosis and bleeding.
- In patients with COVID-19 the risk of both thrombotic and haemorrhagic events needs to be differentially evaluated according with age and endogenous thrombin potential levels.

## Introduction

Coronavirus Disease-2019 (COVID-19), caused by Severe Acute Respiratory Syndrome CoronaVirus-2 (SARS-CoV-2) infection, was first reported in December 2019 in China [1]; since then, it has rapidly become a global pandemic, with a case fatality rate ranging from 2 to 30% according to different age strata and prevalent comorbidities [2, 3]. In particular, an advanced age has been associated with a 300-fold increase in the risk of death [4]. Although patients hospitalized for COVID-19 usually present a respiratory syndrome, they have a hypercoagulable status and are also at higher risk of thrombotic complications [5–7]. To date, the impact of aging on coagulation status and events in patients with COVID-19 patients remains unclear.

Aim of this study was to investigate the association of haemostatic capacity (in terms of endogenous thrombin potential, ETP) on admission with early thrombotic and haemorrhagic complications according to different ages in patients hospitalized for COVID-19.

## Methods

### Study design and population

This is a single-centre, observational, prospective study. Consecutive patients aged > 18 years with pneumonia due to SARS-CoV-2 infection requiring hospital admission in our Institution ("Azienda Ospedaliero-Universitaria Maggiore della Carità" of Novara, Italy) from February 20th to May 29th, 2020 were included. All patients had a nasopharyngeal swab tested positive for molecular detection of SARS-CoV-2 RNA by reverse-transcriptase–polymerase-chain-reaction assay. To avoid any inclusion bias, after evaluation in the Emergency Room, all consecutive patients admitted to low-intensity or medium-intensity COVID-19 wards were enrolled. Patients were included regardless of clinical features at presentation and in-hospital therapies for COVID-19. Patients requiring mechanical ventilation or intensive care unit at hospital presentation were excluded. A consecutive cohort of patients hospitalized during the same time-frame because of infective pneumonia unrelated to SARS-CoV-2 aetiology and no need for intensive care unit at hospital presentation represented the control group. The Institutional Review Board approved the study protocol (IRB code CE 97/20), which was conducted according to the Declaration of Helsinki principles.

### Data collection

We identified eligible patients from hospital administrative data. An electronic case report form was generated, where individual data obtained after daily revision of the clinical records were entered. The data entry was prospectively performed by investigators involved in the patient’s management. A unique pseudonymized code was assigned to each patient. Data including demographic variables, clinical characteristics, medical history, vital signs on admission, laboratory data, medical treatments and clinical events during the hospitalization were collected in all patients. For the purpose of this study, the following in-hospital events were specifically considered: major adverse cardiovascular events (MACE), including cardiovascular death, acute myocardial infarction (assessed as per universal definition) [8], stroke, transient ischemic attack (TIA) or venous thromboembolism (pulmonary embolism detected by computer tomography pulmonary angiogram or deep venous thrombosis assessed by compression ultrasound); bleeding complications, according to the Bleeding Academic Research Consortium (BARC) type 2–5 definition [9]. Briefly, BARC type 3–5 bleeding is classified as fatal bleeding or clinical, laboratory or imaging evidence of bleeding with specific healthcare provider response; BARC type 2 is defined as any clinically overt sign of haemorrhage that is actionable, but does not meet criteria for type 3 to 5. In-hospital death and incidence of severe acute respiratory distress syndrome (ARDS), defined as an acute (≤ 1 week), diffuse, inflammatory lung injury with arterial oxygen tension (PaO_2_) over inspiratory oxygen fraction (FIO_2_) ≤ 100 mmHg [10], were also collected.

### Laboratory analyses

All patients underwent arterial and venous blood sampling upon admission (before starting any anticoagulant treatment). PaO2 was measured in the arterial samples and the following parameters were measured in the venous samples: (a) blood cell count by Sysmex XN-2000™ Hematology System (Sysmex, Kobe, Japan); (b) prothrombin time (PT) and activated partial thromboplastin time (aPTT) by BCS XP (Siemens Healthineers, Marburg, Germany); (c) fibrinogen levels, by Clauss method on BCS XP (Siemens Healthineers, Marburg, Germany); (d) D-dimer levels, by an immuno-turbidimetric assay for the quantitative determination of cross-linked fibrin degradation products in human plasma, performed on BCS XP (Siemens Healthineers, Marburg, Germany); (e) global haemostasis function, by INNOVANCE® ETP test, assessing the ETP of a plasma sample performed on BCS XP (Siemens Healthineers, Marburg, Germany) (see below).

### Measurement of ETP (endogenous thrombin potential)

Global haemostasis function was assessed by ETP, which was measured in platelet-poor plasma using a commercially available assay (Innovance® ETP, Siemens Healthineers, Marburg, Germany). Coagulation activation was initiated by incubation of plasma with phospholipids, human recombinant tissue factor (Innovin®; Siemens Healthcare Diagnostics, Marburg, Germany) and calcium ions in the absence of thrombomodulin. Thrombin generation and subsequent inactivation was recorded by monitoring conversion of a specific slow reacting chromogenic substrate at a wavelength of 405 nm over time. Fibrin aggregation inhibitor prevents the interference of fibrin with the optical detection of the chromophore. A mathematical algorithm was applied to correct the substrate conversion curve. Therefore, the activity of α2-macroglobulin bound thrombin, which has no known biological activity, but is still capable of cleaving small chromogenic substrates, was subtracted from the substrate conversion curve. The corrected reaction curve corresponds to the activity of free thrombin.

**Fig. 1:**
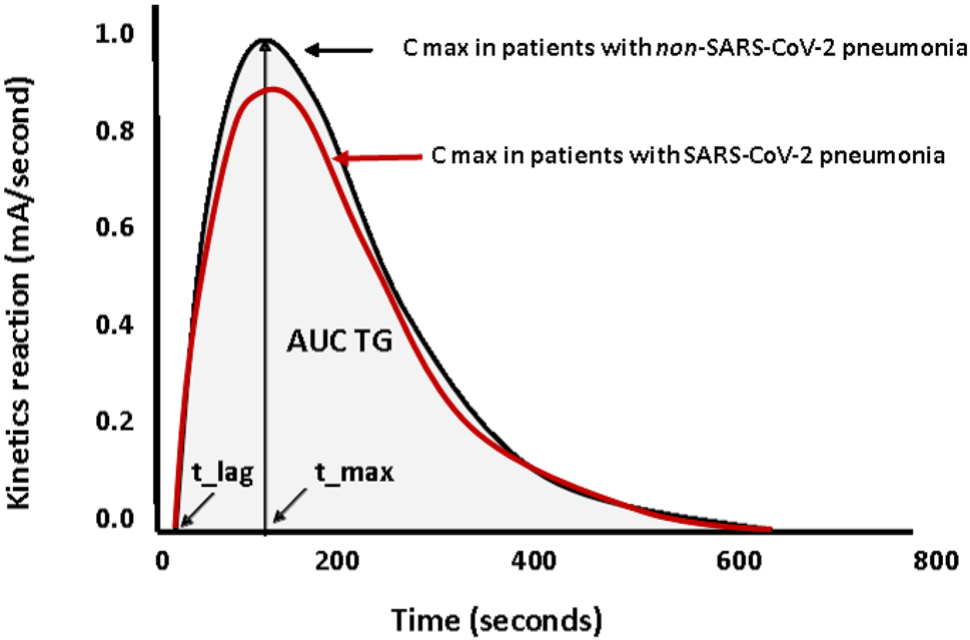
Kinetic curves of ETP (Endogenous Thrombin Potential) in Coronavirus Disease-2019 patients and controls. The ETP value was calculated as area under the thrombin generation curve (AUC TG %); C max is maximum concentration of thrombin; t_lag is lag-time and t_max is time required to reach C max

Formation of the first derivative of the corrected substrate conversion curve corresponds to the thrombin generation curve. The ETP value was calculated as area under the thrombin generation curve (AUC TG) (Fig. 1). Lag time (t_lag) describes the time (seconds) from starting the reaction until thrombin generation is observed. Time to peak (t_max) describes the time (seconds) from starting the reaction until the maximum thrombin generation is observed. Maximum thrombin generation is described by peak height (C max, %). Evaluation of reaction curves as well as computer-assisted calculation of thrombin generation over time used the curve evaluation software Curves, version 1.0 with specification 3.2 (Siemens Healthcare Diagnostics, Marburg, Germany). ETP values are given as percent of normal. Standardization was performed by measuring the ETP standard (Siemens Healthcare Diagnostics, Marburg, Germany) daily in parallel to the patient samples.

### Study endpoints

Study endpoints were the following: (a) to compare ETP levels on admission in COVID-19 patients vs controls; (b) to assess the association between ETP levels on admission and occurrence of in-hospital thrombotic events (MACE) and bleeding complications in the overall population and according to different ages; (c) to evaluate the incidence of adverse events in COVID-19 patients vs controls.

### Statistical analysis

The sample size was not hypothesis-driven, due to the observational nature of the study and the lack of previous specific investigations on the topic. We arbitrarily chose a sample size of at least 50 patients as the minimum number required to provide informative results. Continuous data are reported as mean ± standard deviation, if normally distributed, or median (interquartile range), if not normally distributed, whereas categorical variables are indicated as number (percentage). For the analysis on study parameters in COVID-19 patients vs controls, as well as in patients with vs without adverse events, continuous variables were compared by Student *t* test or Mann–Whitney U test, as appropriate, and proportions by chi-squared test. An interaction test was performed to evaluate the effect of ETP values on adverse outcome (MACE and any bleeding) across different ages by graphing a logit model of age and ETP, as independent variables, and MACE (or bleeding) as dependent variable. Age and ETP values were modelled as continuous variables. Models for the relationship between ETP and outcomes (MACE or bleeding) were plotted at different point values of age (minimum value, 25th, 50th and 75th percentile, maximum value). The analysis was performed by Stata using the command “mfpigen and fplot”(version 16.1 StataCorp College Station, Texas, USA).

## Results

A total of 51 consecutive patients were enrolled, 27 of them with COVID-19 and 24 controls. Among the latter group, the aetiology of pneumonia was obtained in 9 patients by blood culture (3 *Pseudomonas aeruginosa*, 3 *Staphylococcus aureus*, 1 *Staphylococcus epidermidis*, 1 *Klebsiella pneumoniae*, and 1 *Staphylococcus hominis*). Demographic characteristics, comorbidities and medical treatments were similar in COVID-19 patients and controls; regarding laboratory data, lymphocytes count was significantly lower, whereas aPTT and D-dimer levels tended to be higher in the former (Table 1). Thrombin-related parameters (including t_lag, t_max, C max and ETP) were also similar (Table 2). As compared with controls, patients with COVID-19 had significantly higher rates of in-hospital death and severe ARDS, a numerical increase of MACE and comparable bleeding complications (Supplementary Table 1).

**Table 1:** Baseline characteristics in patients with COVID-19 and controls

		COVID-19 patients N = 27	Controls N = 24	p value
*Demographic*				
Age (years)		73 ± 16	71 ± 16	0.632
Male sex		20 (74)	16 (67)	0.562
Body mass index (Kg/m^2^)		29 ± 4	27 ± 6	0.404
*Medical history*				
Arterial hypertension		18 (67)	18 (75)	0.342
Diabetes mellitus		6 (22)	9 (38)	0.086
Atrial fibrillation		8 (30)	6 (25)	0.832
Coronary artery disease		5 (19)	3 (13)	0.765
Stroke or TIA		2 (7)	0	–
Venous thromboembolism		0	2 (8)	–
Active cancer		3 (11)	1 (4)	0.324
Major bleeding		5 (19)	3 (13)	0.285
*Medical treatment*				
Aspirin		6 (22)	5 (21)	0.987
P_2_Y_12_ Inhibitors		3 (11)	3 (13)	0.878
Oral anticoagulant therapy		8 (30)	9 (37)	0.552
Steroids		2 (7)	5 (21)	0.053
Enoxaparin		5 (19)	3 (13)	0.745
*Laboratory findings*	*nv*			
PaO_2_/FiO_2_ ratio	> 300	295 (247–344)	290 (254–324)	0.884
Lymphocytes count (*10^3/μL)	1.00 – 4.50	1.1 ± 0.5	1.5 ± 0.6	**0.018**
Platelet count (*10^3/μL)	150—450	232 ± 80	210 ± 64	0.269
PT (sec)	9.2 – 13.5	11.6 (11.2–12.6)	11.9 (11.2–12.8)	0.650
aPTT (sec)	26.0 – 38.0	36 ± 9	32 ± 4	0.058
Fibrinogen (mg/dL)	180 – 400	531 (440–598)	425 (339–557)	0.124
D-dimer (µg/L)	0 – 500	1542 (619–2262)	935 (382–1529)	0.091
SOFA score		4.5 ± 1.3	4.0 ± 1.2	0.237

Bold value indicates significant at p < 0.05

Data are reported as mean ± standard deviation or median (interquartile range) and number (%)

COVID-19 = Coronavirus Disease-2019; *DVT* Deep venous thrombosis, *nv*  normal values, *PaO*_*2*_*/FiO*_*2*_  arterial oxygen partial pressure to fractional inspired oxygen, *PT*  Prothrombin time, *aPTT*  activated partial thromboplastin time, *SOFA*  Sepsis-related organ failure assessment, *TIA*  Transient ischemic attack

**Table 2:** Parameters of thrombin generation in patients with COVID-19 and controls

	COVID-19 patients N = 27	Controls N = 24	p value
ETP (AUC, %)	93 ± 24	99 ± 21	0.339
C max (%)	106 ± 27	115 ± 17	0.158
t_max (sec)	77 (72–84)	81 (73–96)	0.359
t_lag (sec)	85 (79–100)	91 (82–106)	0.282

Data are reported as mean ± standard deviation or median (interquartile range)

*AUC*  Area under the curve, *C max*  maximal concentration, *COVID-19*  Coronavirus disease-2019, *ETP*  endogenous thrombin potential, *t_lag*  lag time, *t_max*  time to peak

In patients with COVID-19, those with MACE (N = 6) during the hospitalization showed higher D-dimer levels on admission (2299, IQR 2010–2467, pg/mL vs 1126, IQR 568–1616, pg/mL, p = 0.010), lower ETP levels (AUC 86 ± 14% vs 95 ± 27%, p = 0.041) and lower C max (97%, IQR 88–105% vs 115%, IQR 97–128%, p = 0.031) vs those without (Table 3). Patients with haemorrhagic events (N = 4) had higher D-dimer levels (2402, IQR 2039–6654, pg/mL vs 1137, IQR 568–2010, pg/mL, p = 0.0029) and a numerically lower C max (85 ± 21% vs 109 ± 27%, p = 0.093; Table 4). Importantly, 2/3 of COVID-19 patients with in-hospital MACE had also a bleeding complication. A sensitivity analysis dropping patients treated with anticoagulant therapy yielded results consistent with the overall analysis (data not shown).

**Table 3:** Coagulative parameters in patients with vs without in-hospital MACE

	MACE N = 6	No MACE N = 21	p value
PT (sec)	11.3 (11.3–14.6)	11.7 (11.2–12.4)	0.838
aPTT (sec)	34.4 ± 8.4	33.8 ± 6.0	0.828
Fibrinogen (mg/dL)	492 (412–581)	531 (459–598)	0.448
D-dimer (pg/mL)	2299 (2010–2467)	1126 (568–1616)	**0.010**
ETP (AUC, %)	86 ± 14	95 ± 27	**0.041**
C max (%)	97 (88–105)	115 (97–128)	**0.031**
t_max (sec)	74.5 (72–83)	78 (73–84)	0.770
t_lag (sec)	90.5 (81–118)	85 (79–98)	0.414

Bold values indicate significant at p < 0.05

Data are reported as mean ± standard deviation or median (interquartile range)

*aPTT*  activated partial thromboplastin time, *AUC*  Area under the curve, *C max*  maximal concentration; ETP = Endogenous thrombin potential, *MACE*  Major adverse cardiovascular events, *PT*  Prothrombin time, *t_lag*  lag time, *t_max*  time to peak

**Table 4:** Coagulative parameters in patients with vs without in-hospital BARC 2–5 bleeding

	Bleeding N = 4	No bleeding N = 23	p value
PT (sec)	11.4 (11.3–18.6)	11.7 (11.2–12.6)	0.999
aPTT (sec)	32.5 (31.3–46.1)	35.8 (32.3–37.9)	0.973
Fibrinogen (mg/dL)	492 (392–558)	531 (440–611)	0.339
D-dimer (pg/mL)	2402 (2039–6654)	1137 (568–2010)	**0.029**
ETP (AUC, %)	82 ± 16	95 ± 26	0.337
C max (%)	85 ± 21	109 ± 27	0.093
t_max (sec.)	74 (68–115)	78 (73–84)	0.834
t_lag (sec.)	82 (80–121)	86 (79–100)	0.973

Bold value indicates significant at p < 0.05

Data are reported as mean ± standard deviation or median (interquartile range)

*aPTT*  activated partial thromboplastin time, *AUC*  Area under the curve, *BARC*  bleeding academic research consortium, *C max* maximal concentration, *ETP* Endogenous thrombin potential, *PT* Prothrombin time, *t_lag*  lag time, *t_max*  time to peak

**Fig. 2:**
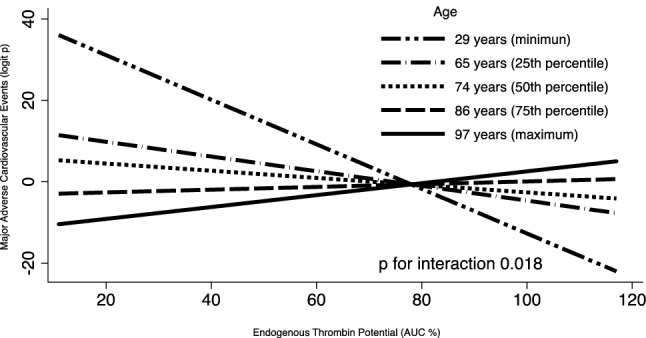
Interaction plot of Endogenous Thrombin Potential (ETP) on Major Adverse Cardiovascular Events (MACE) across minimum age, 25th, 50th and 75th percentile of age, and maximum age. For low ETP levels (i.e. AUC 20%), the logit function of MACE in patients aged 65 and 85 years is 10.0 and − 2.2, corresponding to a probability of 0.999 and 0.100, respectively. For high ETP levels (i.e. AUC 120%), the logit function of MACE in patients aged 65 and 85 years is − 7.0 and 0.2, corresponding to a probability of 0.001 and 0.550, respectively. *AUC* Area under the curve

Among the COVID-19 cohort, the analysis across different ages showed a linear increase of MACE logit function by decreasing ETP values on admission in patients aged 65 years (25th percentile). Conversely, no relationship between MACE logit function and ETP values was estimated in patients with age 85 years (75th percentile; p for interaction 0.018) (Fig. 2). In controls, there was no interaction between age and incidence of MACE by ETP levels.

**Fig. 3:**
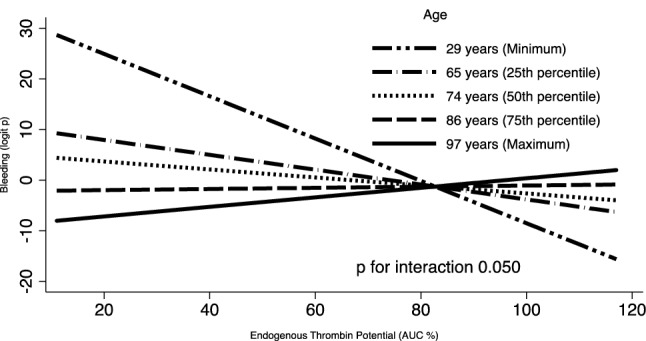
Interaction plot of Endogenous Thrombin Potential (ETP) on any bleeding complication across minimum age, 25th, 50th and 75th percentile of age, and maximum age. For low ETP levels (i.e. AUC 20%), the logit function of bleeding in patients aged 65 and 85 years is 6.9 and -1.7, corresponding to a probability of 0.999 and 0.154, respectively. For high ETP levels (i.e. AUC 120%), the logit function of bleeding in patients aged 65 and 85 years is − 6.9 and − 1.2, corresponding to a probability of 0.001 and 0.230, respectively. *AUC* Area under the curve

Moreover, in COVID-19 patients, similarly to what observed for MACE, the analysis across different ages showed in patients aged 65 years a linear increase of bleeding logit function by decreasing ETP values on admission. Again, no relationship between bleeding logit function and ETP values was estimated in patients aged 85 years (p for interaction 0.050) (Fig. 3) and no interaction between age and incidence of bleeding by ETP levels was observed in controls.

## Discussion

In this prospective study on a consecutive cohort of patients hospitalized for COVID-19, we found an interaction between age and ETP levels on admission for both in-hospital MACE and bleeding complications. In particular, in younger patients there was an inverse relationship between ETP values and adverse outcome (i.e. the lower ETP levels, the higher the event risk). Notably, if ETP levels were reduced, the estimated risks of MACE and bleeding in patients aged 65 years were 10-fold and 6-fold higher, respectively, than in those aged 85 years. Moreover, in patients aged 65 years having the lowest ETP values, there was an estimated 100-fold increase in both MACE and bleeding vs those with similar age, but highest ETP levels. Importantly, such age-dependent pattern was not observed in patients with pneumonia unrelated to SARS-CoV-2 infection.

Several reports have described an increased occurrence of arterial and venous thrombotic complications in COVID-19, either macrovascular and microvascular [11–14]. Such an involvement of the entire circulatory system suggests a unique, systemic disease mechanism. Indeed, a multifaceted interaction between coagulation abnormalities (coagulopathy), platelet hyper-reactivity and endothelial dysfunction has been described in COVID-19 pathobiology [15]. COVID-19 coagulopathy is characterized by a pro-thrombotic state with high levels of D-dimer, fibrinogen and its degradation products [12, 16], and reduced endogenous fibrinolysis [17, 18]. However, data on thrombin generation yielded controversial results, as some studies here reported low levels of prothrombin fragment 1 + 2 and thrombin–antithrombin III complexes, whereas other investigations described increased ETP levels [19–21]. The present study found comparable ETP levels on admission in patients with COVID-19 and non-SARS-CoV-2 pneumonia. However, this finding might be affected by sample size, use of an early, single ETP measurement and a non-high risk profile of the study population (i.e. lung disease not requiring mechanical ventilation).

Various investigations have evaluated age-related variations of coagulative parameters in healthy individuals. In particular, previous studies demonstrated a direct relationship between ETP values and aging, with such evidence pleading age-adjusted range values [22]. Similarly, an increment of 10 mg/dL in fibrinogen levels per each decade of age can be expected in healthy subjects [23]. To date, no study has investigated the age effects on coagulation and adverse events in COVID-19. We explored such issue with a special focus on thrombin potential. Our abovementioned results support a COVID-19 pathology where low ETP levels reflect an unbalanced coagulation with elevated thrombin consumption in a pro-thrombotic milieu. In particular, this appears to be prevalent in younger patients, in agreement with other investigations on different settings, where an inverse relationship between ETP levels and adverse outcome was demonstrated [24–26]. Conversely, we can hypothesize a different pathogenesis for hypercoagulable state and higher risk of thrombotic events in older COVID-19 patients, in whom an impaired endogenous fibrinolysis may be prevalent; this is in line with previous evidence demonstrating an age-dependent increase of molecules antagonizing thrombus lysis (i.e. plasminogen activator inhibitor) [23].

Besides thrombotic events, bleeding is also a relevant cause of morbidity in patients hospitalized for COVID-19 [27]. Notably, previous data indicated that increased levels of D-dimer on admission were also predictors of bleeding complications in COVID-19 [27]. Our findings confirm the association between increased D-dimer values and in-hospital bleeding. Importantly, we observed an interaction between age and ETP levels on the likelihood of bleeding. In particular, in the range of lowest ETP levels, younger patients were at higher risk than older ones. Indeed, a consistent haemostatic pattern has been previously described in other viral infections, though with a typical haemorrhagic presentation (such as Dengue fever), where a reduced ETP was observed [28].

Lastly, the results of the present study support an unbalanced coagulation equilibrium towards both thrombosis and bleeding with increased thrombin consumption in COVID-19. This is confirmed by the fact that 2/3 of patients with in-hospital MACE had also a bleeding event. Indeed, a similar haemostatic unbalance (with reduced ETP levels) has been described in patients with disseminated intravascular coagulation, where the consumption of coagulation factors exceeds the liver synthesis and is associated with both thrombotic and haemorrhagic complications [29].

Our investigation has to be considered in light of its limitations. Data collection on medical history and comorbidities was mainly based on patients' reports and therefore is potentially biased. A gap between in vitro and in vivo ETP measurement may exist [30], as in vitro testing presents differences compared to in vivo testing. Moreover, no specific reference ranges for ETP levels are available in the setting of COVID-19. Finally, our findings concern patients with COVID-19 without need for intensive care unit or mechanical ventilation at hospital presentation, and whether they are also applicable to more severe clinical patterns is unknown.

In conclusion, the present study indicates that the risk of both early thrombotic and haemorrhagic events is increased in younger patients with low ETP levels on admission for COVID-19. This pattern was not present in patients with non-SARS-CoV-2 pneumonia. These findings may have implications for the management of COVID-19 patients. According to our data, younger patients with low ETP values should receive during hospitalization a strict surveillance and a close monitoring for changes in clinical features and for abnormalities of coagulative parameters (i.e., prothrombin time, fibrinogen, D-dimer, platelet count, fibrin degradation products) to prevent and early diagnosing serious adverse events [31]. However, our results are hypothesis-generating and require specific mechanistic studies.

## Supplementary Information

Below is the link to the electronic supplementary material.

Supplementary file1 (DOCX 28 kb)
