# Supplementary material for: Interaction between thrombin potential and age on early clinical outcome in patients hospitalized for COVID-19
Source: J Thromb Thrombolysis. 2021 Jun 10;52(3):746–53. doi: 10.1007/s11239-021-02497-1 (PMC8190736; doi:10.1007/s11239-021-02497-1)
Supplement: Supplementary file 1 — Supplementary file1 (DOCX 28 kb) [file 11239_2021_2497_MOESM1_ESM.docx]

**Supplementary Table 1.** In-hospital complications.

|  | **COVID-19 patients**  **N=27** | **Controls**  **N=24** | **p value** |
| --- | --- | --- | --- |
| All-cause death | 8 (30) | 0 | **0.004** |
| MACE | 6 (22) | 1 (4) | 0.061 |
| Cardiovascular death | 4 (15) | 0 | 0.050 |
| Myocardial infarction | 1 (4) | 1 (4) | 0.932 |
| Stroke or TIA | 1 (4) | 0 | 0.341 |
| Venous thromboembolism | 1 (4) | 0 | 0.341 |
| Severe ARDS | 15 (56) | 4 (17) | **0.003** |
| Bleeding complications | 4 (15) | 2 (8) | 0.473 |
| BARC type 3-5 bleeding | 3 (11) | 2 (8) | 0.739 |
| BARC type 2 bleeding | 1 (4) | 0 | 0.341 |

Data are reported as number (%).

ARDS= Acute respiratory distress syndrome; BARC= Bleeding Academic Research Consortium; COVID-19= Coronavirus Disease-2019; MACE= Major adverse cardiovascular events; TIA= Transient ischemic attack
